# Schistosome infection and its effect on pulmonary circulation

**DOI:** 10.21542/gcsp.2019.5

**Published:** 2019-03-31

**Authors:** Ghazwan Butrous

**Affiliations:** Professor of Cardiopulmonary Sciences, Medway School of Pharmacy, University of Kent, UK and University of Greenwich, Central Ave, Gillingham, Chatham ME4 4BF, Kent, UK

## Abstract

Schistosomiasis is the most common parasitic disease associated with pulmonary hypertension. It induces remodelling via complex inflammatory processes, which eventually produce the clinical manifestation of pulmonary hypertension. The pulmonary hypertension shows clinical signs and symptoms that are not distinguishable from other forms of pulmonary arterial hypertension.

## Introduction

**Figure 1.**
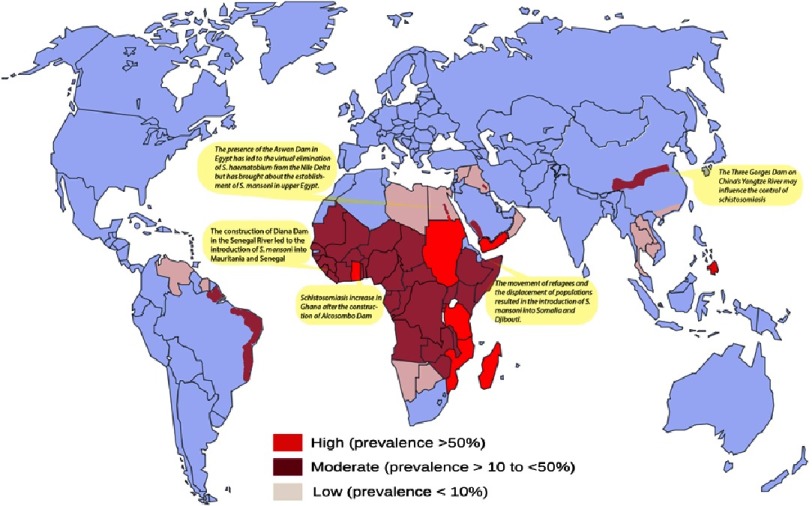
A world map showing the geographical distribution of Schistosomiasis with its approximate prevalence. The yellow bubbles are examples of incidences that increase the prevalence of Schistosomiasis (see text).

Schistosomiasis (bilharziasis) is a disease caused by infection with blood flukes of the genus *Schistosoma*. The disease is transmitted to human by contact with infested water with special fresh water snails, which act as intermediary for the life cycle of the parasite (see below). The condition remains one of the most prevalent parasitic infections in the world. An estimated 240 million people are affected in 78 countries, and close to 800 million are at risk. Africa, and in particular sub-Saharan Africa, is the main affected area with about 80% of the infected global population (Figure 1). Other areas affected include Eastern Mediterranean region, South Americas and Western Pacific regions. The condition is not present Europe or North America^1–4^. Current statistics suggests that almost 120 million people have symptoms, and 20 million have severe illness with hepatic or urological involvement, and a subset, reported between 5–54%, have evidence of pulmonary involvement^1–3,5,6^. As such, along with malaria, schistosomiasis is one of the most important of all human parasitic diseases. It continues to be a global public health concern in the developing world.

Schistosomiasis is a chronic infection that is rarely recognized in its early stages. Chronic schistosomiasis is the most prevalent form of the disease in regions endemic for schistosomiasis, due to repeated exposure and re-infection. In general, a child’s initial infection occurs by age 2 years. There is a noticeable increase in intensity during the next 10 years with the burden of re-infection. Thus, the highest prevalence and intensities of infection occur in young adolescents, that is 60–80% of school age children and 20–40% of adults. The prevalence can persist among sub populations of adults who have frequent contact with water during their daily activities—e.g., laundry, bathing, fishing, or washing animals or cars^7,8^. Typically, the disease is largely a rural problem. Urban foci can be found in many endemic areas, and sex-related patterns vary in relation to behavioural, professional, cultural, and religious factors^9^. The disease disables men and women during their most productive years. It is estimated to be as high as 29 million disability-adjusted life years (DALYs)^10^.

Over the last 50 years there has been an international control program with success in many parts of the world^11^. The World Health organization in May 2012 urged member states to eliminate schistosomiasis with a dual strategy for the control of schistosomiasis: a strategy for morbidity control adapted to the public health context in high burden areas, and a strategy to consolidate control in areas where a low endemic level has been reached and elimination may be feasible^12,13^. The preventive chemotherapy (i.e., periodic large-scale administration of the anti-schistosomal drugs to school-aged children and other high-risk groups is the main practical approach^13–15^. Currently the main anthelmintic drug used in the treatment of schistosomiasis is praziquantel^16^, discovered in the mid-1970s. From a public health point of view it requires adequate monitoring of current mass drug administration programs. Alternative compounds like Artemisinins, Oxamniquine or Furoxan have been tested, but none so far is completely satisfactory and are not in general use^17,18^.

Schistosomiasis vaccine development has been an uphill battle, and there are still several hurdles to overcome in the future. There are currently few schistosomiasis vaccines in clinical trials^19^.

Further important measures like the elimination of intermediate host snails, and the prevention of human contact with water containing infected snails, can help to prevent the transmission^18,20–22^.

Despite these efforts, the control was not universal because of the economical and developmental projects and environmental changes^12^ that affect the snail’s habitat, one of the crucial determinants of transmission.

For example, the development of new irrigation programs leads to a remarkable redistribution of schistosomiasis worldwide, e.g., the construction of Diana Dam in the Senegal River led to the introduction of schistosomiasis into Mauritania and Senegal.

Construction of the Egyptian Aswan High Dam in 1960s led to the virtual elimination of *S. haematobium* from the Nile Delta, but has brought about the establishment of *S. mansoni* in upper Egypt^14,23–26^.

The Three Gorges Dam, currently being built on China’s Yangtze River is between two areas where schistosomiasis is endemic. The Chinese Ministry of Health is currently evaluating the potential effect of the dam on schistosomiasis transmission.

The movement of refugees and the displacement of populations resulted in the introduction of schistosomiasis into Somalia and Djibouti. Furthermore, there are reports of resistance to praziquantel^27–31^. Thus, despite major efforts and advances in control and substantial decreases in morbidity and mortality, schistosomiasis continues to spread to new geographic areas^12,31^.

## Discovery of Schistosomiasis

**Figure 2.**
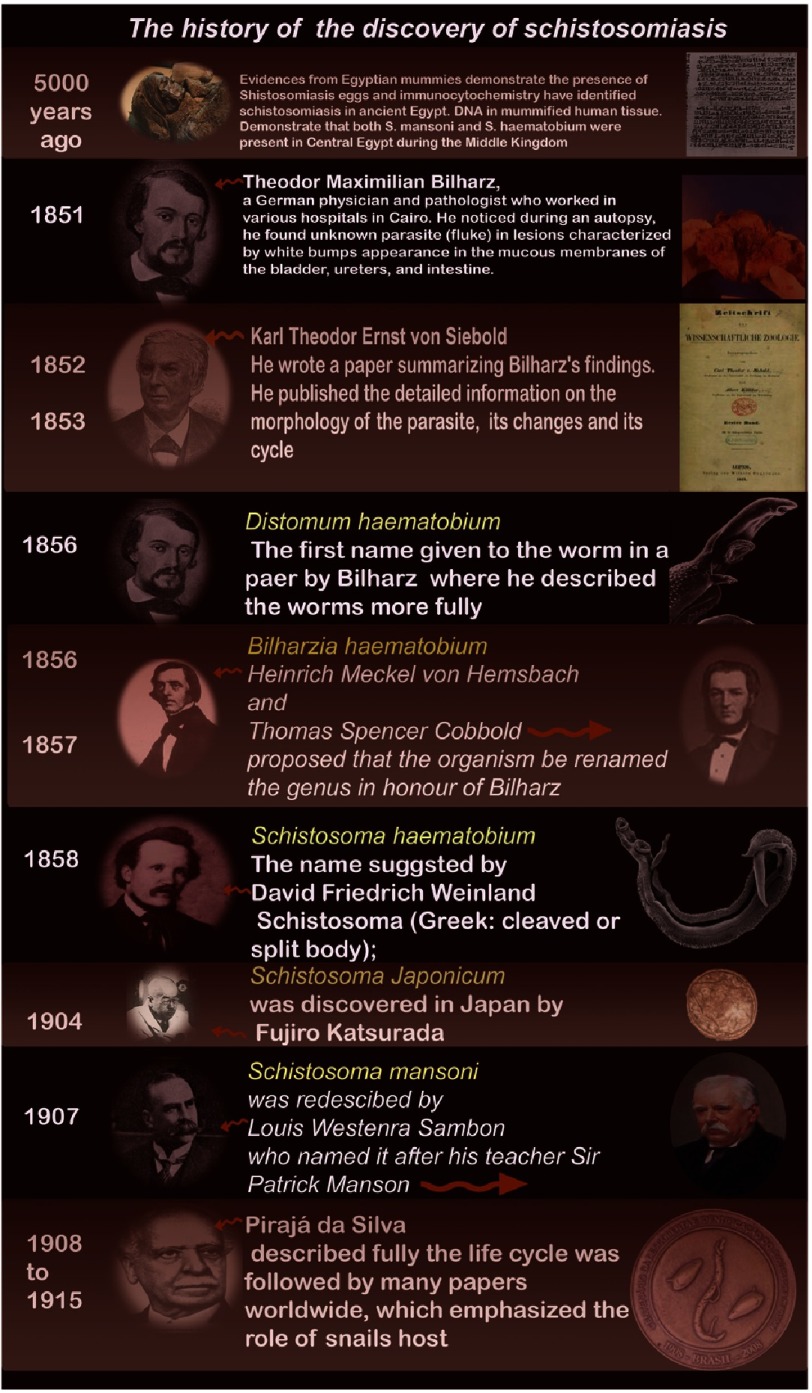
Infographic history of discovery of schistosomiasis from ancient time till the commencement of the 20th century.

Schistosomiasis was one of the oldest diseases known to mankind. It was discovered in 5000-year-old Egyptian mummies. Its characteristic symptoms are described in early Egyptian papyri^32–34^. Theodor Maximilian Bilharz, a German physician and pathologist, who worked in various hospitals in Cairo in the middle of the 19th century, noticed the worm in white bumps on the mucous membranes of the bladder, ureters, seminal glands and intestine in 1851^35^. His discovery was the beginning of a new era for tropical parasitology and finding preventive and curative measures. Other discoveries and observations followed in the second half of the 19th century and the beginning of the 20th century. Figure 2 is an infographic of the main milestones of the early discovery of the schistosomiasis.

## The Parasite

The etiologic agents of the schistosomiasis are the blood flukes, which belong to the genus *Schistosoma of* the class of Trematoda of *the* phylum of Platyhelminthes (flatworms). It is white-greyish cylindrical body approximately 1 to 1.5 cm in length. It can survive for up to 30–40 years with an average of 3 to 10 years in their human hosts. The schistosomes have separate sexes and live in the blood vessels of the abdominal cavity.

The male holds the slender female fitted into the gynaecophoric canal in a continuous monogamous embrace. She produces eggs and he fertilizes them. The worm has two suckers, a complex tegument, a blind digestive tract, and reproductive organs. They feed on blood cells and globulines, which they digest in their intestinal tract. They have no anus and cannot excrete waste products, so they regurgitate waste into the bloodstream which can be useful for blood-based and urine-based diagnostic assays^36,37^. The worm takes energy via anaerobic metabolism, which serves mainly for the movements of the male schistosomes and the egg production of the females^38,39^.

Schistosoma is invisible to human immune defences and, like many other parasites, they show a unique ability to manipulate the immune system^40,41^. This invisibility can be attributed to some schistosome genes that resemble human ones. Furthermore, the unusual covering known as the tegument^37^, a second external body layer, may contribute to the parasite’s ability to hide from the immune system. The tegument provides ample protection to the worm as it migrates through human blood. The nature of tegument function is still under investigation; some thought that this outer coat could acquire human molecules from the blood, covering the parasite’s own molecules and making them invisible to immune surveillance^42^. This subject is of interest to vaccine designers, because the targets of most successful vaccines are proteins or other molecules that appear on the outside of a pathogen.

Schistosome populations are genetically heterogeneous^43,44^. Genetic diversity and inbreeding were higher in East compared to West African populations, and differ between schistosome species. This has implications for the transmission of schistosomiasis and the potential establishment of drug resistance^45^. Genomic characterisation of human schistosomes can help establish epidemiological patterns of transmission, including insights into interspecies hybridization among some schistosome species. The new release of SchistoDB (http://SchistoDB.net) provides a rich resource of genomic data for genus *Schistosoma*^46^.

The three most important human schistosomes are *Schistosoma mansoni,* S. *japonicum,* and *S. haematobium.* They live within either the perivascular (*S haematobium*) or mesenteric (*S mansoni, S japonicum*, and others) venules. There are several other species of lesser importance which infect humans in Africa and Asia^4^.

*S. mansoni* has the widest geographic distribution. *S. mansoni* remains endemic in Africa, Arabian Peninsula, parts of Brazil, Venezuela, the Caribbean and Puerto Rico. It is found in primates and rodents, but humans are the main reservoir. Adult worms live in the portal system of the liver and the small venules of the lower ileum and colon. The prepatent period for the infection is approximately 6 weeks. In chronic schistosomiasis, eggs will accumulate in the liver, walls of the intestine and rectum. Fewer eggs will be found in faeces. The eggs in the faeces help in the reliable diagnosis of the infection. Rectal biopsies may also be useful in diagnosis.

*S. japonicum* is found in China, Indonesia, and the Philippines. It has been virtually eliminated in other countries of Southeast Asia, including Japan. Infection rates remain high in China and the Philippines because in these regions the infection is with a zoophilic strain of the parasite (i.e., it is also maintained in animal hosts including water buffaloes, pigs, dogs, cats, and wild rodents)^47^. The adult worms live in mesenteric veins, and the prepatent period is 5 to 6 weeks. In addition, the surface of the egg frequently has faecal debris adhering to it, which can make the eggs difficult to see or recognize. Rectal biopsy may be an important diagnostic tool when faecal examinations are negative.

*S. haematobium* occurs in some parts of Africa, Egypt, Middle East, Iran, and the Arabian Peninsula. It causes urinary schistosomiasis. Adult worms reside in the venous plexuses of the bladder, and eggs that are laid move through the wall of the bladder and pass in urine. In chronic infections, accumulation of eggs in the bladder wall can lead to the bladder and ureter pathology. Haematuria is frequently present with this infection and diagnosis is made by finding eggs in urine sediment. Eggs can sometimes be found in faeces and in the wall of the rectum as well as in the bladder.

Other less common species can also infect human like *S. mekongi*, which like S. japonicum is a zoophilic strain. It is mainly seen in countries bordering on the Mekong River, specifically Laos and Cambodia. *S. intercalatum* is a human schistosome that occurs in Zaire, Gabon, Cameroon, and the Central Africa Republic. The eggs of this species are found in faeces, have a terminal spine, and thus must be differentiated from *S. haematohium* eggs. Eggs are usually larger than those of *S. haematobium*, measuring 140–240 µm long by 50–85 µm wide.

## The Different Forms of the Parasite and its Life Cycle

**Figure 3.**
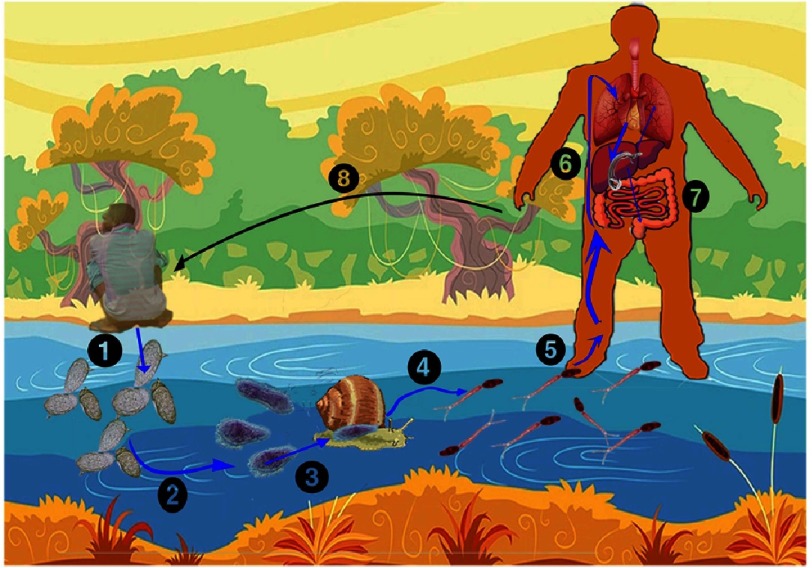
Schistosoma life cycle. (1) Eggs passed to the fresh water with faeces or urine (depends on the schistosomiasis species ); (2) when eggs hatch Miracidia will be released in the fresh water; (3) Miracida enter intermediary freshwater snails (depends on the schistosomiasis species, see text; (4) after certain gestating period Cercaria are released from the snails to the freshwater; (5) Cercaria enter human venous system (6) after entering the venous system the worn undergo transformation and changed and reside in the target organs (7). The worm will produce many eggs. Some organs trap eggs causing pathological changes, other are released with faeces or urine, and the cycle continues.

Schistosomes have a complex life cycle (Figure 3) involving both a snail intermediary and a vertebrate definitive host. Understanding the schistosome life cycle and the parasite’s movement between intermediate (snail) and definitive hosts (man or other mammalian) is fundamental to understand the pathological changes, the control and elimination of human schistosomiasis.

**Figure 4.**
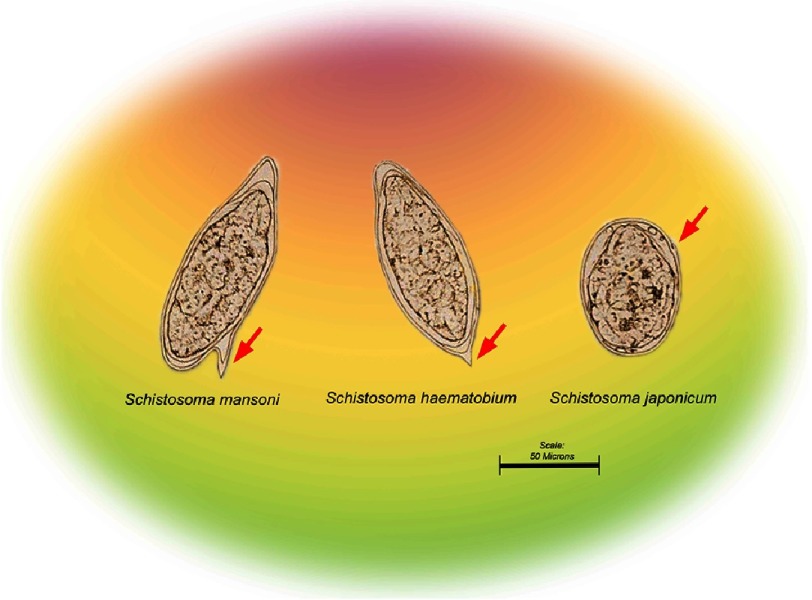
Eggs from the main three human Schistosoma parasite, showing the size and the location of the spine (red arrows) which is one of the main species differentiators.

Eggs are thin-shelled that lack an operculum and contain a miracidium^48^. The size, shape, and the location of the spine, varies according to the species (Figure 4). *S. mansoni* eggs measure 114–175 µm long by 45–70 µm wide. *S. japonicum* eggs are round and measure 70–100 µm long by 55–65 µm wide with a small, inconspicuous spine, which frequently is not seen. The thin-shelled eggs of *S. haematobium* measure 112–170 µm long by 40–70 µm wide and have a terminal spine.

Some eggs allow make their way into faeces, or urine in the fresh water and thus out of the body to continue the life cycle. Eggs can stay viable for up to 7 days with low osmolality; but with bright sunlight the eggs hatch and release miracidium^48,49^. These larvae (about 150 µm long) are non-feeding and swim rapidly (2 mm/s for about 6 hours) using cilia attached to epidermal plates to locate a compatible snail-intermediate host. Swimming behaviour is photokinetic, and possibly chemokinetic towards snail components. The sensory terebratorium (apical papilla) facilitates attachment to the snail surface. They penetrate the snail by release of proteases from their lateral and apical glands with the support of the mechanical movement. Thus, schistosomes require a snail host to complete the life cycle they develop. Only freshwater snails are involved with mammalian transfer. Each of the schistosomes utilizes specific and different snail intermediate hosts. The most important snail species are

1. Biomphalaria snails for *S. mansoni* (purely aquatic snails)
2. Bulinus snails for *S. haematobium* (purely aquatic snails)
3. Oncomelania snails for *S. japonicum* (spend part of their lives in the mud where they can survive low temperatures).

Inside the snail host, the miracidium sheds its ciliated plates and becomes a post-miracidium. A new syncytial tegument is formed and the larva differentiates into a mother sporocyst that subsequently produce asexually germ-cell daughter sporocysts^50^. These migrate to the hepatic and gonadal tissue of the snail. The mechanisms by which the parasite evades the snail-host defines response are not currently well understood but are likely to be multi-factorial. Within 4-6 weeks, these daughter sporocysts metamorphose into cercariae; which also multiply asexually, producing large number of cercariae to complete its life in the snail. Under the stimulation of light, typically between 10am and 4pm when the sun is high and human water contact most frequent, hundreds of free-swimming, fork-tailed cercariae leave the snail^51,52^.

The cercariae is a free-living, non-feeding form with a length of about 325 µm. There are many similarities of cercariae of mammalian schistosome and it is difficult to differentiate them. The body and bifurcated tail are a muscular temporary locomotor organ of a cercaria that is enveloped with a single continuous syncytial tegument, covered by a carbohydrate-rich glycocalyx. They swim backward (tail-first) using intermittent bursts of activity to locate a suitable definitive host^53^. The swimming continues for several hours (up to 72 h) seeking the skin of a suitable definitive host until cercarial glycogen reserves are depleted.

Water turbulence, shadows and certain skin chemicals influence host finding (human or animals). Ciliated sensory papillae exist and are thought to facilitate host detection. The acetabulum (ventral sucker) is well developed. Various glands exist that contain multiple enzymes including proteases and are important for host penetration and cercarial function^51,54^. Once the definitive host in penetrated, the bifurcated tail of cercariae is shed and they become schistosomules^55^. This new form passes through the epidermis and dermis before exiting via the blood or lymphatic vessels.

There seems to be a difference in the way different schistosome species move through the human skin. *S. mansoni* and *S. haematobium* move through the human skin layers slower than *S. japonicum*. It takes about 48 hours for the majority of *S. mansoni* and *S. haematobium* schistosomula to reach the dermis, and 72 hours for these species to be found near the dermal blood vessels.

Successful transformation is considered essential for parasite survival. They then embark on a complicated journey, first penetrating the host dermis and venule wall and entering the circulation via the venous or lymphatic vessels. Next, they migrate through the pulmonary capillaries (lung schistosomula), which is the greatest obstacle (not the skin). A proportion of schistosomula entered the alveoli, from where they do not recover or enter the systemic circulation later^56,57^. The successful schistosomula of *S. mansoni* then pass to the hepatic portal system and begin to blood feed, pair up, and migrate to the portal vessels and mesenteric venules. For S*. haematobium*, the worms finally pass to the vesical venules around the bladder where they reside. In these final locations, schistosomula grow rapidly and the tegument matures. Males grow larger than females and display higher mitotic activity. Development of the sex organs occurs after approximately 3 weeks (for *S. mansoni*) or 9 weeks for *S. haematobium*. The females produce hundreds (*S. mansoni* and *S. haematobium*) to thousands (*S. japonicum*) of eggs per day^52,58^ and continue producing eggs throughout their lifetime.

## Pathological Effects of Schistosome Infection

The cardinal feature of schistosomiasis is not the mature worm, which has evolved immune evasion mechanisms that allow them to remain incognito within the bloodstream^59^, but by the highly antigenic egg-associated pathology that is central to the morbidity and mortality. When eggs are trapped in various tissues or the areas of venous drainage (liver or lung), they cause the formation granulomatous inflammation around the parasite within maximal 8 weeks post-infection. This process is wholly dependent on CD4^+^ T lymphocytes^59,60^. The exact mechanism by which eggs pass across the endothelium, intervening tissues and mucosal epithelium remains unknown, but clearly is dependent upon the host immune response, as egg excretion does not occur in animals that are immunocompromised.

**Figure 5.**
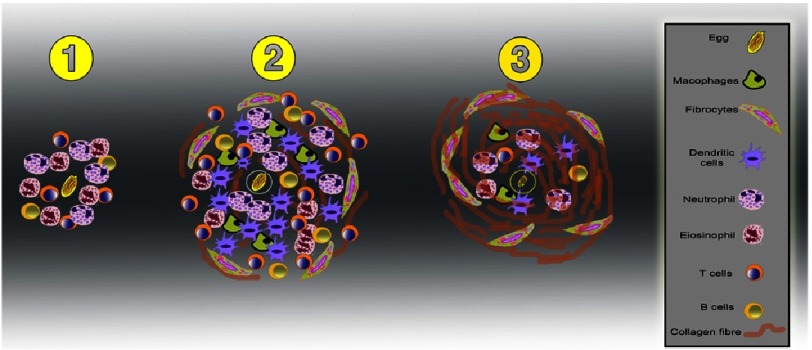
Schematic representation showing the development and cellular composition (right panel) of the granuloma around the Schistosoma eggs. The proportion and types of cells, which comprise granulomas varies depending on the species of infecting schistosome. The process of granuloma development is driven by the egg’s antigens and its growth by cytokines and chemokines released by T lymphocytes. Granuloma formation begins with the initial recruitment of macrophages, eosinophils, and neutrophils around the entrapped egg (Panel 1). These cells cause an inflammatory reaction causing the recruitment of more cells and the development of the granuloma size (Panel 2). Panel (3) shows the final stage of, resolve the granuloma once the antigenic stimulus (mainly the eggs) has been destroyed, resulting in deposition of fibrils of chromatin and collagens, and subsequent collagen degradation and remodelling that contribute to the final tissue fibrosis.

The granuloma is the aggregation of mononuclear inflammatory cells, macrophages resembling epithelial cells, lymphocytes, multinucleated giant cell eosinophils, and plasma cells, monocytes and T cells in addition to eosinophils, lymphocytes and mast cells (Figure 5). The proportion of cells differ in different organs around the egg, with the liver being more sensitive than the lung in its response^61,62^. Furthermore, granuloma formation around *S. mansoni* eggs is much more intense than that around *S. japonicum* eggs^63,64^. It prevents diffusion of the toxic egg substances and eventually destroys them. It also leads to tissue destruction and may cause fibrosis after many years^64–66^.

## Hepatic Pathology

Liver disease develops secondary to entrapment of eggs in portal venules (<50 mm in diameter) and is initially presinusoidal^6^. Eggs remain viable in the liver for about 3 weeks, with immunological resection to egg antigens started with type 1 helper (Th1) in response, evolving to a dominant Th2 immune and the peri-ovular granulomas development. Subsequently there will be recruitment of eosinophils, associated with angiogenesis, and these will lead to the fibrogenesis of the liver^67,68^.

These pathological changes can be scattered in the liver parenchyma and in periportal spaces, resulting in periportal fibrosis, and the development of “Symmers pipe stem fibrosis”^69,70^. In addition to the development of granuloma and fibrosis, other pathological changes have been noticed, including hepatovascular endothelium proliferation, smooth muscle cell dedifferentiation, hyperplasia of elastic tissue, and the presence of several collagen isotypes. Distortion of the small and medium-sized capillaries and the appearance of small vessels sprouting from the largest portal veins, but with preservation of arterial, liver lobular architecture and ductal structures, were also observed^68^.

Later, periportal collagen deposition leads to a gradual obstruction of venous blood flow, so that often the entire portal vein wall is destroyed, transforming an open circulation to a partly closed one, leading to decreased blood flow^71,72^. This is associated with hepatomegaly, splenomegaly and portal hypertension^64^. Approximately 5–10% of patients chronically infected with schistosomiasis develop the hepatosplenic form of the disease (collectively known as hepatosplenic schistosomiasis)^73^. Clinically, portal hypertension in the first 25 years of life may cause oesophageal varices and ascites^74–77^. In general, the natural history of portal hypertension is closely related to the number of eggs deposited in the liver^78^. However, unlike the fibrosis with hepatic cirrhosis, Symmers pipe stem fibrosis may show occasional degradation and subsequent signs of portal hypertension (as splenomegaly and oesophageal varices) can progressively disappear^79,80^.

Eggs that are retained in the gut also induce inflammation, ulceration and colonic polyposis^64^ which may cause diarrhoea. A small increase in colorectal cancer has also been reported^64,65^. These pathological changes usually do not interfere with liver function until the passage of many years of heavy infection.

## Lung Pathology

The eggs can occasionally become trapped in the lungs. It is thought that the development of portal-systemic collateral circulation leads to a hyperdynamic circulatory state, contributing to an increased flow and sheer stress to the pulmonary circulation^81^. It also facilitates the shunting of eggs from the liver to the lungs, thus increasing the immunological burden on the lungs and pulmonary circulation^56,82^.

**Figure 6.**
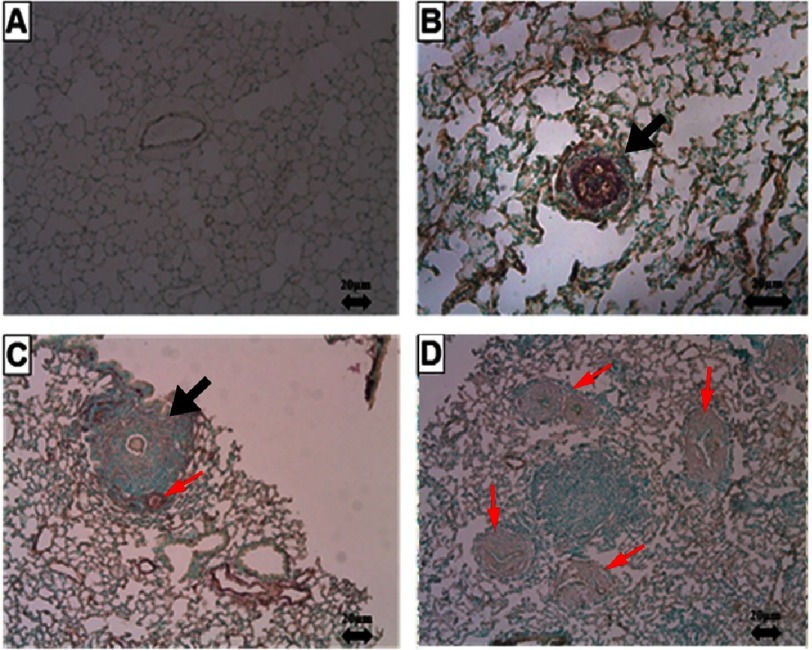
Schistosomiasis induced granuloma in the lung (mouse model 12 weeks after infection with Schistosoma cercaria). Panel A shows control normal lung tissue Panel B is showing remodelled arteriole inside a granuloma (black arrow). Panel C shows a peripheral granuloma surrounding an egg (black arrow), with a remodelled arteriole (red arrow) in the periphery of the granuloma. Panel D shows various remodelled vessels nearby a granuloma (red arrows). Staining (alpha-smooth muscle actin and von Willebrand Factor). Scale bar = 20 µm. Source: the author laboratory.

Like the liver, the highly antigenic eggs will develop an immunological reaction that leads to the development of granuloma, and subsequent remodelling of pulmonary arterioles (Figure 6). Severe intimal, medial, and adventitial hypertrophy and proliferation of a plethora of inflammatory cells occurs in the pulmonary vasculature, which contributes to the development of pulmonary hypertension^74,82–86^. There was also evidence that eggs are destroyed completely and more rapidly in the lungs than in the liver^87^.

**Figure 7.**
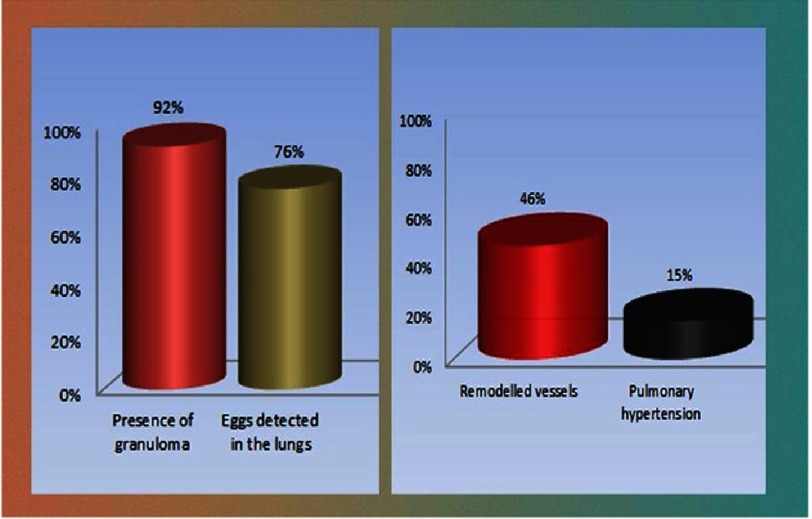
The mouse model of 12 weeks after infection with Schistosoma cercaria, showed that more than two third of the mouse could be infected with eggs and presence of granulomas (left panel). However, the right panel shows that only half of the lungs show some form of remodelling pulmonary arteries; but only 15% showed a feature that can be presented as pulmonary hypertension (mainly severe remodelling and presence of right ventricular hypertrophy). (Adopted from the author laboratory data originally published in 90).

Autopsy studies from Brazil of 78 patients with hepatosplenic schistosomiasis showed that 72% had periovular granulomas in the lung, but only 18% showed obliterative endarteritis endothelial cell hyperplasia, fibrin depositions and vascular hypertensive changes^82^. These are followed by complex fibrin thrombus and revascularization, congestion and dilatation of focal blood vessels, with angiogenesis which results in plexiform lesions^84,88,89^. In the author’s laboratory the same trend^90^ was noticed in mice infected with cercariae for 12 weeks. Seventy-nine percent of the lungs harvested from these animals contained evidence of granulomatous changes. Remodelled pulmonary vessels were seen in 46% of the lungs, and were observed in close proximity to the granuloma, but only 15% or less showed evidence of severe right ventricular hypertrophy (Figure 7).

Thus, granuloma due to chronic schistosoma infection in the lung may cause nearby vascular changes. These include endothelial cell dysfunction, loss of endothelial barrier integrity, proliferation of endothelial cells and fibroblasts, thrombi, intimal fibrosis, and formation of new endothelial lined channels ‘plexiform-like lesions’^87,91,92^ with some similarities to the pulmonary vascular remodelling reported in idiopathic pulmonary hypertension^93–97^ (Figure 6).

In one recent observation in patients with pulmonary hypertension due to schistosomiasis, dark pigments were frequently observed adjacent to pulmonary vascular lesions. The origin of these pigments is still unknown and is thought unlikely to be parasite-derived^98^.

Curative treatment can promote reversion of the periovular granulomatous lesions formed in the alveolar tissue but not always. The changes in the arterial and arteriolar lesions were defectively repaired, with segmental vascular fibrosis, narrowing, and angiomatoid changes remaining for up to 120 days after treatment. This persistence of pulmonary vascular diseases after complete parasitic infection resolution was originally observed in experimental animals by de Almeida and Andrade in 1983^87^. Portal vein ligation–induced portal hypertension, but after *S. mansoni* infection, the pulmonary vascular abnormalities persisted even after the eggs were eradicated and granulomas were resolved after treatments.

The same observations were seen in the liver, where remodelling of the vasculature remained after the resolution of the infection by chemotherapy^79^. Furthermore, it was noticed in mice models infected with schistosomiasis eggs that the granulomas contained degraded eggs as well as egg antigens and macrophage lysosomes^98^. On the other hand, the lung tissue of patients who died of pulmonary hypertension due to schistosomiasis showed granulomas and remodelled pulmonary vasculature as well as plexiform lesions, but no visible eggs or significant egg antigen^98^.

Schistosomiasis vasculopathy is not rare^90^, but frequently seen in patients with Symmers fibrosis^91^. There were suggestions this is possibly due a mechanical obstruction by the ova or worm of the pulmonary arteries leads to a focal arthritis^99,100^, but most investigators believe it is due to some sort of allergic reaction, intense immunological and inflammatory reaction^101–103^. The portal hypertension may contribute to the pathological changes by opening the collateral vessels that help schistosoma eggs to escape to the lung tissue. This will increase the antigen burden on the lung and thus aggravates pulmonary hypertension^104^.

Aggravation of pulmonary hypertension has also been described after the surgical treatment of schistosomiasis-associated portal hypertension with a shunt procedure^105,106^. Pulmonary hypertension has been seen in 2–12% of patients with portal hypertension in general^107^, and in 21% of the cases with portal hypertension due to hepatosplenic mansonic infection^106^.

## Immunology and inflammatory role on the pathogenesis of pulmonary hypertension due to schistosomiasis

Inflammation plays a central role in both the causes and consequences of the pathogenesis of pulmonary hypertension due to schistosomiasis^73,92,108–112^. T cells, B cells, mast cells, macrophages, and dendritic cells, as well as inflammatory cytokines and chemokines, are found in the lungs of patients with pulmonary hypertension, in various parts of the remodelled small pulmonary arteries, and in plexiform lesions^113–116^. The athymic mouse model failed to respond to the schistosome eggs with a granulomatous response in either the liver or the lung^117^.

Crosby *et al.* described marked perivascular inflammation, and showed a time-dependent increase in the levels of circulating cytokines, when the Th1 cytokine (mainly TNF-α and IL-1β) peaked at 12 weeks, while Th2 cytokines (IL-10, IL-13, IL-6, and IL-4) peaked at 17 weeks^92^. Furthermore, the macrophage marker (CD68 and CD45) lymphocytes cells increased. The degree of pulmonary vascular remodelling correlated with lung egg burden and various inflammatory cytokines^92^.

Evidence of inflammatory markers is accumulating in schistosomiasis pulmonary vascular diseases, and elevated levels of inflammatory cytokines predict survival in idiopathic and familial pulmonary arterial hypertension^118^ –but the role of inflammatory mediators in general is not universal. There are differences in results from patients and animal models. A recent study showed that pulmonary arterial hypertension patients exhibited significant elevations in TNFα, IL-6, and MCP-1, versus healthy participants; while this was not the case in animal models of monocrotaline, after SU5416 alone after SU5416 plus hypoxia^119^.

The granulomas, are the dynamic structures that require the persistent stimulation of activated macrophages and cytokines from other inflammatory cells and in particular the T cells^61,120,121^.

Initially there is a disorganized aggregation of cells (the pre-granulomatous stage) followed by a gradual accumulation of mononuclear and eosinophils around the freshly deposited egg. As the granuloma matures, dendritic and stellate cells, tissue macrophages, epithelioid cells and fibrocytes begin to appear at the periphery, leading to gradual degeneration of the schistosoma eggs and appearance of fibrocytes and collagen fibres. This results in the reduction of the granuloma size, which contains only pigmented macrophages and may exhibit hyalization of collagen fibres, whereas the egg is typically is integrated and may become calcified^122–125^.

The cellular and chemical factors controlling granuloma formation are considerable, incompletely understood, and show some difference in among different schistosoma species. It also varies according to the strain, species, age, gender, duration or intensity of infection, genetic predisposition, co-infection, reinfection, the chronological changes of the maturing egg, and other environmental factors^58,126^.

**Figure 8.**
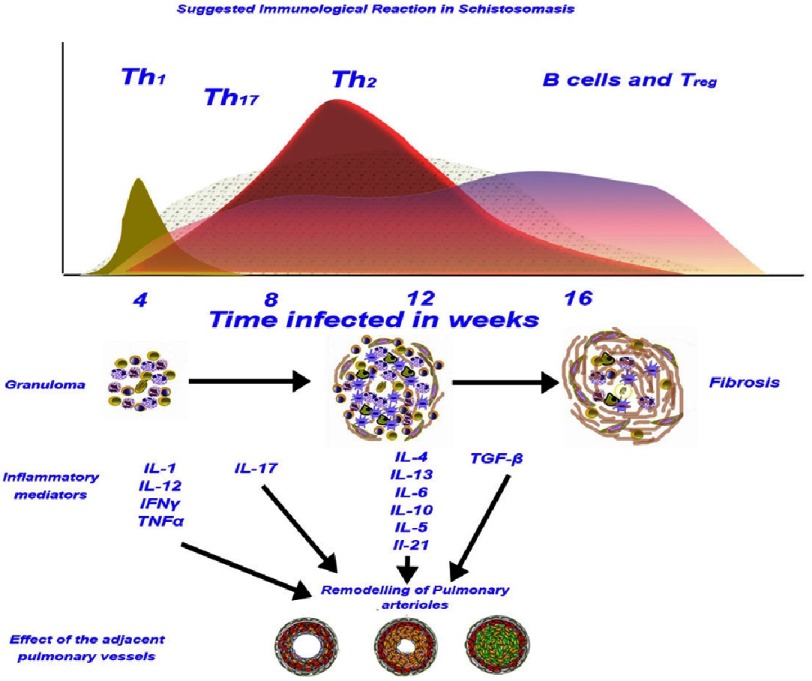
The upper panel shows a hypothetical diagram of a simplified chronological sequence of the T helper cells after the tissue is exposed to schistosome eggs. Th1 phase is a short and immunologically aggressive initial stage, which starts a longer Th2 phase. The 2 phase helps in suppressing the Th1 activities reducing its the aggressive nature. At the same time a less understood Th17 started and remained for a longer period (see text for more details). During Th2 the most active cytokines (see the lower panel (inflammatory mediators) help in further development and maturation of the granuloma (second panel (granuloma). The major remodelling process of the pulmonary arteries can happen during this stage (Th2) (see the bottom panel) although other staff (ex Th1 and Th17) can play a part. This will be followed the appearance of Treg cells and the predominance of B cells, with the resolution of the granuloma and in most occasion the development of fibrosis.

As mentioned above the early immunological response involves CD4^+^ and CD8^+^ T helper (Th) cells, and thus generalised type 1 response dominates (Figure 8). It is also associated with an expansion in the frequency of cytotoxic T cells (Tc1) and Th1 causing an increase in the expression of the Th1 proinflammatory cytokines IL-1, IL-12, tumour necrosis factor (TNFα) and interferon gamma (IFNγ).

These mediators can be measured in the plasma and infected tissue and in the peripheral-blood mononuclear cells^58,127^. They may modulate the release of chemokines such as CXCL2, CXCL5, CXCL9, CXCL10, CXCL11, CXCL22, CCL3, CCL7, and lymphotactin (XCL1)^128^.

By about 6-8 weeks after infection the cytokine response changes to type 2 with an increase in the numbers of Th2 cells and a which dramatic downregulate of Tc1 cells via apoptosis process and reducing the effect of type 1 inflammatory responses. The increase in Th2 activities enhances growth of the granuloma, the mobilization of other cells like eosinophils and eosinophils, and increases the process of collagen deposition (Figure 8)^67,129^.

The polarising inflammation toward Th2 response and the Th-1/Th-2 cross-regulation and the growth of granuloma formation is mainly mediated by IL-4 and IL-13. This results the production of high levels of Th2 cytokines such as IL-4, IL-5, IL-10, IL-13, IL-21, IL-17, IL-31 and the synthesis of immunoglobulin E (IgE), AMCase, Ym1, and RELM α^58,129–134^. These different cytokines have various roles. For example, some of the cytokines can enhance fibrosis, whereas others can prevent it.

Th2 response starts to down-modulate by around 12 weeks, and is associated with the reduction of the granuloma size, which is thought to be induced by IL-10 and TGFβ^58,121,132,135^. Furthermore, Th17 response has also been reported in various schistosoma species^136,137^. The cytokine IL-17 has been shown to significantly contribute to the development of a severe granulomatous^138^.

B cell distribution and their absolute numbers of B cells in the spleen, lymph nodes and blood increased dramatically during the early Th2 phase of infection, and remained high throughout the chronic infection. B cells also play a central role in mediating the transition from Th1 to Th2 responses and in the regulation of the granulomatous response during acute and chronic infection.

B cell-deficient mice developed significantly smaller hepatic granulomas^139^. L-10, a cytokine implicated in plasma cell development, is important for the role of B cells. It was noticed that IL-10 receptors blockade precipitated the development of portal hypertension together with the shunting of eggs, resulting in the development of severe pulmonary pathology and the accumulation of parasite eggs in the lungs and heart^140–142^ – confirming the vital role of the B cells in during schistosoma infection, and probably the development of pulmonary vasculopathy^143^.

Extrinsic apoptosis pathways (mainly via TNF path, but in particular FAS path) play an important role in the regulation of the immunological reaction to schistosome a eggs and the development of the granulomas, thus mitigating further host damage^144–147^.

The first apoptosis signal (Fas) receptor (also known as Apo-1 or CD95) binds the Fas ligand (FasL), a transmembrane protein part of the TNF family^148,149^. They trigger the death of lymphocytes and thus are fundamental to regulating T-cell and B-cell lymphocyte maturation, receptor repertoire selection, and homeostasis^150^.

Thus the granuloma maturation, and consequently their effect on pulmonary vascular disease remodelling, can be influenced to a certain degree by the role of FAS-FASL pathways^138^. But until now there have been very few studies on the direct role of extrinsic apoptosis on schistosomiasis-induced pulmonary vascular disease. For example, soluble FAS is significantly higher in schistosomiasis pulmonary hypertension compared with those in patients with cor pulmonale resulting from chronic obstructive pulmonary disease^151^. Furthermore, soluble FAS (SFas) is shed from the cell surface through the action of certain enzymes like metalloproteinases^152^. SFas can antagonize FAS pathway (FAS-FASL pathways) and thus inhibit apoptosis, which may contribute to the remodelling process of schistosome pulmonary vascular disease. This subject is still being studied and needs further investigation.

### Th-1 proinflammatory mediators

Earlier studies showed that, in general, patients with pulmonary hypertension demonstrated TNFα serum levels within the normal range, but had increased serum levels of IL-1 beta and IL-6 in severe primary pulmonary hypertension. These observations suggest a role for proinflammatory cytokines in primary pulmonary hypertension and remodelling of the pulmonary vessels in certain animal models^153^. Proinflammatory cytokines such as IL-1 and TNFα may contribute to the vasoconstriction tone, or as mediators of oxidative stress and endothelial dysfunction^154^. Other mechanisms are explained below for each of the specific cytokines of Th1 type.

#### Interleukin-1

Interleukin-1 (IL-1) is a potent proinflammatory cytokine. The best-known members are IL-1α, IL-1β, and IL-1Ra (receptor antagonist). Various cells can produce this cytokine, such as macrophages, lymphocytes, endothelial cells, vascular smooth muscles cells, and fibroblasts^155^. It can be a positive or negative mediator of cell apoptosis.

IL-1 is released in the early stages of schistosoma infection, and may participate in the pathogenesis of some forms of pulmonary hypertension. Treatment with human IL-1 receptor antagonist inhibits the development of pulmonary hypertension in monocrotaline, but not in chronically hypoxic rats^156,157^; the latter probably due to the effect of lL-1 as proapoptotic during hypoxia^158^.

It was noticed in patients with pulmonary hypertension that increased levels of IL-1 increased unadjusted hazard of death^159^. IL-1β impairs the cyclic AMP pathway in response to prostacyclin analogues in pulmonary artery smooth muscles cells, resulting in an imbalance of the vasoactive components that may lead to pulmonary hypertension^160^. The combination of TGF-β1, IL-1β and TNF-α, induced morphological and phenotypic changes consistent with of endothelial-to-mesenchymal transition with a dominant effect by IL-1^96^.

#### Interleukin 12

Interleukin 12 (IL-12) is mainly produced by dendritic cells, macrophages, neutrophils, and some B-lymphocytes^161,162^. IL-12 binds to the IL-12 receptors (IL-12R and IL-12Rβ2). Its main function is the differentiation of the naïve T cells into Th1 cells and help to produce Th1 cytokines like interferon-gamma (IFN-γ) and tumour necrosis factor-alpha (TNF-α)^161–163^. It also reduces IL-4 mediated suppression of IFN-γ. IL-12 blocks the formation of new blood vessels. Elevated IL-12 cytokine expression inhibits secondary granuloma formation, fibrosis and blocks the formation of new blood vessels in mice pre-sensitized with schistosoma eggs^164,165^.

#### Interferon-gamma

Interferon-gamma (IFNγ) is the primary cytokine secreted by CD4 Th1 cells and natural killer (NK) CD8 cytotoxic T lymphocyte, mainly by the effect of IL-12 (see above) and Interleukin 18 (IL-18 ) on T-cell receptors ( TCR )^166^. IFNγ activates specific receptors that orchestrate various signalling selectivities within the JAK/STAT pathway. IFNγ also plays an important role in differentiation of T helper 1 (Th1) cell population via a positive feedback mechanism. The IFN-γ level is elevated after *Schistosoma* infection^167,168^. Th1 helper cells in the granuloma aggregate around the macrophages and secrete IFN-γ and TNF, demonstrating the critical role of IFN-γ (and also TNF - see below) in the development of the granuloma function and endothelial damage^169–173^.

IFN-γ also induces a potent vasoconstriction on human pulmonary smooth muscles cells by helping in the release endothelin-1^174^. Thus, IFN-γ exhibits pleiotropic effects that could similarly influence differential immune regulation in early pulmonary arterial hypertension development. Other types of interferon (mainly type I interferon) were also associated with pulmonary hypertension and mediate signalling mechanisms that cause further remodelling of the pulmonary vessels and right ventricle on increased serum ET-1 in hypoxia model^175^.

#### Tumour necrosis factor

Tumour necrosis factor (TNFα) is a cytokine involved Th1 inflammation. It is produced many cell types chiefly mainly activated macrophages, but also CD4^+^ lymphocytes, natural killer cells, neutrophils, mast cells, eosinophils, and neurons. TNFα works on various signalling pathways like the activation of NF-*κ*B, Mitogen-Activated Protein Kinases (MAPK) pathways and enhancement of apoptosis mainly via the extrinsic apoptosis pathways. Pulmonary hypertension developed spontaneously in transgenic mice overexpressing TNF *α*^176^. TNFα plays an important role in the development of pulmonary hypertension, even though the concrete mechanisms remain unknown, but the evidences are mounting. Recently it was demonstrated that TNFα reduces *BMPR2* expression in vascular cells^177^. It induces morphological and phenotypic changes consistent with endothelial-to-mesenchymal transition, which contributes to the remodelling process. In addition the monocrotaline-treated rats that were injected with Etanercept (a TNFα antagonist) showed protection from development of pulmonary hypertension^178^. An increased release of TNFα has been implicated in triggering the progression of the disease^159^. Furthermore, the high plasma levels of TNFα increased unadjusted hazard of death^159^.

### Th-2 proinflammatory mediators

Chronic schistosoma infection, like that of other helminths, drives a strong Type-2 inflammation, thought to be largely mediated by a CD4^+^T helper 2 (Type -2 (Th2)) response, while suppressing the Type-1 response. This Th2 response, triggered by egg-derived antigens, includes cytokines IL-4, IL-5, IL-10 and IL-13. Some Type-2 cytokines, mainly IL-4 and IL-13, play critical roles in activation of signalling pathways within cells that regulate cascades of immunological response of transformation from Th1 to Th2^179^. This signalling may then regulate downstream targets involved in inflammation, maturation and dissolution of granulomas and later fibrosis and vascular remodelling^124,180^.

#### Interleukin 4

Interleukin 4 (IL-4) is a compact globular folded protein that binds to a cellular receptor (IL-4Rα)^181^. It is one of the major Th2 cytokines and is produced mainly by mast cells, basophils natural killer T (NKT) cells, and Th2 cells themselves (positive feedback loop)^182–184^. IL-4 has pleiotropic effects on many cell types^185^. Its effects depend on binding to a receptor complex, resulting in a series of phosphorylation events mediated by associated kinases. Stat 6 plays a central role in exerting IL-4-mediated biological responses that lead to various cellular functions. These include cell growth, resistance to apoptosis, and of gene activation and differentiation. It also influences transcription (JAK/STAT) signalling cascades, which may contribute to allergic responses^179,181,185–187^. Some of its effect is closely related to the functionality of IL-13^186^ (see below).

IL-4’s main roles in the regulation of the immune response include:

1. It is one of the principle cytokines in Th-2 response. It induces differentiation of naive helper T (Th0) to Th2 cells (CD4^+^ Th2 cells)^185,188^.
2. Proliferation of activated B-cells and the differentiation of B cells into plasma cells, thus influence IgG1 and IgE production.
3. It is a potent survival factor for B cells and induces the expression of class II MHC (major histocompatibility complex), in B cells^185,189^.
4. IL-4 decreases the production of Th1 cells, macrophages, IFN-gamma, and dendritic cell and IL-12^189^; thus, plays a protective role during schistosomiasis by controlling the production of other cytokines.
5. IL-4 induces the so-called “alternative macrophage activation”^190^.

Thus IL-4 has been shown to play a major part in the development of granuloma and fibrosis in around *S. mansoni* eggs in the liver, but its influence was more on the growth of granuloma formation in the lungs of the same animals^191,192^. IL-4’s role on the remodelling of pulmonary vascular diseases has been documented and the severity of remodelling may be correlated with high levels of IL-4^193^. However, this not conclusive yet as Crosby et al noticed that there is no correlation between RV/LVIS and any individual cytokine^92^.

The athymic rat model treated with the SU5416 (VEGF receptor 2 inhibitor used in some animal models to induce pulmonary hypertension) also showed elevated levels of IL-4 in lung tissue extracts, which may enhance the number of mast cells, B cells, and macrophages in perivascular infiltrates, despite the deficiency of T-celIs^194^.

IL-4 signalling may play a significant role in hypoxia-induced mitogenic factor (HIMF) induced lung inflammation and vascular remodelling^195^. Despite all these observations, the role of IL-4 on the remodelling process and pulmonary hypertension is still uncertain and needs further investigation.

#### Interleukin 13

Interleukin 13 (IL-13) is a pleiotropic cytokine which is produced by many cell types, including Th-2 lymphocytes, mast cells and unique innate lymphoid 2 cells (Nuocytes)^196^,^197^. IL-13 has been shown to be a crucial mediator of Th-2 dominant immune responses to variety of helminth infections^58,67^ and is a major inducer of fibrosis^198,199^.

Its role is closely related to the cytokine IL-4 and may work in tandem^186^. It is a prime effector molecule, while IL-4 may be of immunoregulatory importance^200^. IL-13 and IL-4 binding varies in different human cell types, depending on co-expression of IL-4R and IL-13R which form complexes and share common subunit(s)^201^. IL-13 activates the receptors IL-13Rα1, which hetrodiamerizes, and IL-4Rα (which does not by itself bind IL-13)^201^. IL-13 has another receptor subunit, IL-13Rα2, which binds IL-13 exclusively with high affinity. This receptor appears to lack a signalling motif and exists in both membrane-bound and soluble forms. IL-13Rα2 initially was thought to be a “decoy” receptor for IL-13 (i.e., , one that binds IL-13 without producing biological effects)^202^.

The complex with “decoy” receptors is thought to be a selective and powerful inhibitor of IL-13-induced inflammation, remodelling, and profibrotic effects^203^. Thus the signalling mechanism of this cytokine are similar to that of IL-4; that is enhancement of Janus kinase (JAK) activity and subsequently activation of transcription (STAT6) that can initiate transcription of target genes which contribute to production of various inflammatory, profibrotic and regulatory mediators.

IL-13 has many biological effects of interest in our review, such as:

1. It can be involved in induction of TGF-β (see below) by monocytes and macrophages and subsequently contributing in the process of fibrosis^180^.
2. IL-13 regulates endothelial vascular cell adhesion molecule-1 (VCAM-1) and elicits a spectrum of responses in vascular endothelium expression^204,205^.
3. Production of matrix metalloproteinase-9^206^.
4. IL-13 acts as a potent antiproliferative, but not proapoptotic, factor for pulmonary artery smooth muscles.
5. IL-13 potently down-regulates endothelin-1 production in the lung^193^.

Increased expression of both IL-13 and its receptors in small pulmonary arteries of patients with idiopathic pulmonary arterial hypertension has been reported^207^. IL-13 was elevated in the plasma of patients with systemic sclerosis with pulmonary arterial hypertension compared with patients without pulmonary arterial hypertension^208^. IL-4/IL-13 signalling in pulmonary tissue from individuals who died of schistosomiasis associated- pulmonary hypertension, underscoring the potential clinical relevance. Pulmonary hypertension were observed in mice exposed to inhaled Aspergillus^193^ and in IL-13–overexpressing mice developed pulmonary hypertension^209^. However different observations were reported by Hecker et al, that IL-13 signalling suppressed proliferation of human pulmonary artery smooth muscle cells *in vitro*^207^. These authors^207^ noticed that pulmonary expression of the IL-13 decoy receptor (IL-13Rα2) was up-regulated relative to that of the IL-13 signalling receptors IL-4R and IL-13Rα1 in patients with pulmonary hypertension and some animal models - suggesting the important regulatory role of IL-13 in the remodelling process.

IL-13 has also recently been implicated as a regulator of pulmonary artery remodelling induced by a Th2 immune response^193^, suggesting that this cytokine may contribute to the pathogenesis of pulmonary vascular diseases in schistosomiasis. It is an important mediator of granulomatous and vascular responses in the murine model schistosomiasis infection and consequently the development of pulmonary hypertension^111^. Crosby et al noticed IL-13 has been implicated in pulmonary arterial muscularization and IL-13 (but not IL-4) stimulated migration of mouse pulmonary artery smooth muscle cells^92^.

**Figure 9.**
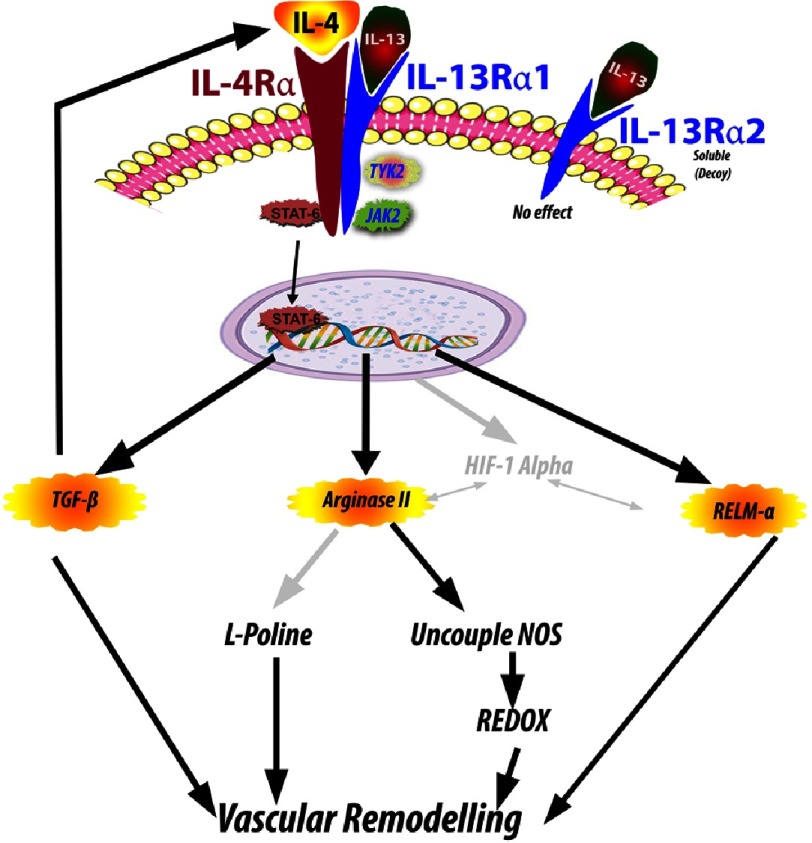
Diagrammatic representations of IL-4 and IL13 showing the interaction of IL-13 and IL-4. These cytokines can affect the remodelling via three possible pathways; that is via TGF-β, Arginase II or RELMα pathways (see text for more details).

The signalling mechanism of IL-13 in schistosomiasis pulmonary vascular pathology is still far from fully appreciated (Figure 9). Graham et al observed that IL-3 contribution to the pulmonary vasculopathy may be due to the increase in TGF-β1 synthesis and IL4 and IL13 to be necessary for TGF-β activation via its activation of STAT6^110,210^. They noticed an increased expression of the canonical TGF-β target phospho-Smad2/3 in the pulmonary intima, media and adventitia. Blockade of TGF-β signalling prevented the development of experimental schistosoma-induced pulmonary hypertension^212,213^ Others observed that TGF-β signalling is higher in mice exposed to *Schistosoma* and patients who have died of schistosoma-induced pulmonary hypertension^212^.

Thus, it is most likely that remodelling process by IL-13 and IL-4 and dependent TGF-1 showed positive feedback mechanism^212,214^ . Furthermore, these two cytokines can also contribute to the remodelling process via other signalling process. Cho et al found that that arginase 2 (see Box 1) is a critical downstream mediator of IL-13-induced pulmonary hypertension^209^ via enhancing proliferation of vascular cells and expanding extracellular matrix.

Arginase may also compete with nitric oxide synthase for the mutual substrate, arginine, leading to decreased bioavailability of NO^215^, recognized as the major mediator of the maintenance of vascular homeostasis. However, it can help in uncoupling of nitric oxide synthase and generates more reactive oxygen species rather than NO, leading to an increase in reactive oxygen species (ROS), which ultimately causes vascular oxidative stress and inflammation contributing to the development of vascular diseases, contributing further to the remodelling process^216,217^.

Further, some of the arginase products includes polyamines and L-proline, which are known to stimulate cell growth and differentiation and collagen synthesis^218,219^. Recently, an ongoing investigation was based on the hypothesis that hypoxia-inducible transcription factor HIF1α plays a critical role in IL-13-stimulated proliferation pulmonary vascular smooth muscles cells (Figure 9). IL-13Rα2-Arg2-HIF1α pathway regulates mitochondrial metabolism of the mitochondria of proliferation pulmonary vascular smooth muscles cells. This hypothesis still needs further confirmation^220^.

The third observation was the that the downstream product of the IL-4 and IL-13 signalling pathways in the lung may lead to an increase in the numbers of RELM-α positive cells that were seen in the borders of remodelled pulmonary arteries in mice models^193^ .

BOX 1 Arginase is a manganese-containing enzyme ubiquitous to all domains of life. It is the focal enzyme of the urea cycle hydrolysing l-arginine to urea and l-ornithine and plays a role in the regulation of nitric oxide synthesis. It is present mainly in two forms Arg1, a cytosolic protein, mainly expressed in hepatocytes as a key enzyme in the urea cycle and Arg2 is a mitochondrial protein that is expressed in a variety of extra hepatic tissues) are the key enzymes in l-arginine metabolism that convert l-arginine to l-ornithine and urea. This enzyme helped conversion of Arginine to NO (in this case the term Coupled NOS is used). Increase in the activity of Arginase 2 reduces the availability of Arginine and hence enhances the eNOS-uncoupling (see BOX 2 for eNOS). Therefore, resulting in the formation of Reactive oxygen species instead of NO (the term uncouple eNOS is used here because that is, uncoupling of NADPH oxidation and NO synthesis, with oxygen instead of l-arginine as terminal electron acceptor)^151^.

BOX 2 Endothelial nitric oxide synthase (eNOS) is the critical enzyme in the maintenance of vascular pressure by producing NO that plays a major role in the relaxation of smooth muscle surrounding the arterioles^151–156^

#### Interleukin 6

Interleukin 6 (IL-6) is a pleiotropic cytokine which is produced by professional antigen-presenting cells (APC) such as B cells, dendritic in addition to T-cells, macrophages mast cells, and muscle cells^221,222^. IL-6 exerts its biological activities through a unique receptor system. These receptors are composed of a membrane-bound IL-6 receptor and two gp130 molecules. The activation of this receptor leads to the so-called **classical signalling pathway**. This pathway may have some anti-inflammatory responses and is restricted to hepatocytes, monocytes, macrophages, and lymphocytes.

The other receptor system is the **trans-signalling pathway,** whereby IL-6 binds to a soluble IL-6 receptor (sIL6R) found in bodily fluids such as urine and blood. That consequently activates membrane gp130 initiating signalling cascades allowing this to affect larger types of cells and some important biological effects, such as growth and pro-Inflammatory activities. The signalling cascade if IL-6 involved JAK/STAT3^223–225^.

IL-6 also influences several aspects of the T cell immune response and act as a survival factor for T cells. It promotes the differentiation of naive CD4^+^ T cells into IL-4 –producing effector Th2 cells.

IL-6 regulates IL-4 gene expression mainly by activating nuclear factor of activated T cells (NFAT)^226–228^. It also inhibits Th1 differentiation. This generalization is common in IL-6 but detailed signalling pathways differ markedly between various tissues. For example, IL-6 signalling in macrophages, dependent upon activation of the NFkB signalling pathway, other tissues show TGF-β/SMAD3 or MAPK signalling pathways. Thus, IL-6 has a complex mechanism - being proinflammatory, anti-inflammatory as well as profibrotic function. It can play bidirectional role for example up regulation of IL-6 at the early inflammatory and more regulatory role throughout^229^.

Both classical and trans-signalling pathways^230^ were noticed in the smooth muscle layer of remodelled vessels in human and experimental models, as in monocrotaline and chronic hypoxia rats^222,231,232^. The ablation of IL-6 can be protective in the chronic hypoxia model^222^. It is unclear whether IL-6 affects pulmonary vascular remodelling by directly targeting vessel-wall cells, or by indirect effects mediated by inflammatory cells. IL-6 may affect vascular remodelling via several complex mechanisms^222^. Its effect is still elusive and far from clear. Davies et al^233^ suggested that IL-6 production (and probably IL-1) in the pulmonary vasculature was a result of chronic down regulation of SMAD3 and may mediate several inflammatory mediators. IL-6 induction may become uncontrolled, contributing to the development of pulmonary hypertension ^233,234^. IL-6 promotes the development and progression of pulmonary vascular remodelling through pro-proliferative antiapoptotic mechanisms, increased the number of platelets^232,235^. Some clinical studies have shown that IL-6, like many other proinflammatory cytokines, increases in patients with pulmonary arterial hypertension and is associated with hazard of death^118,159,236,237^; though the French Network of Pulmonary Hypertension suggests that IL-6 was not significantly associated with increased hazard of death^159^.

**Figure 10.**
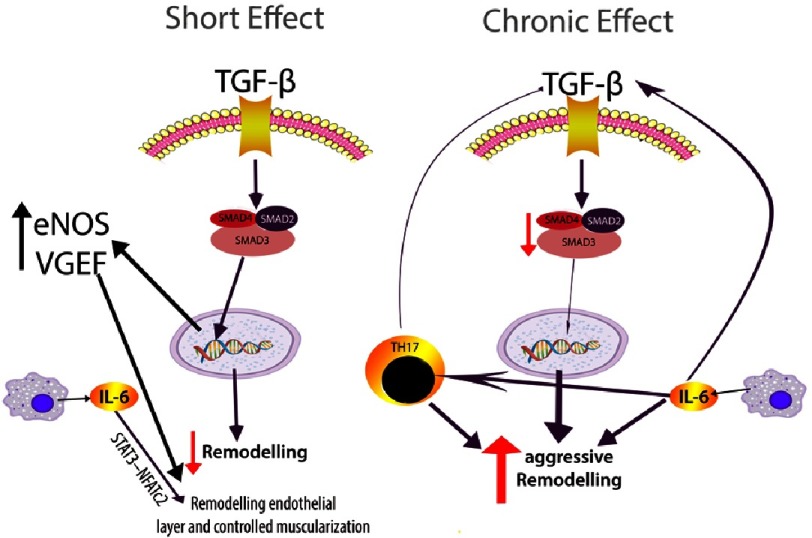
Current understanding of the role of IL-6 and its interaction with TGF-β in the pathogenesis of schistosomiasis pulmonary vascular pathology. TGF-β can have a complex mechanism as described in the text. In the early stage or short-term exposure to TGF-β (left panel) can stimulate via SMAD3 eNOS and VGEF and thus reduce the remodelling process, and IL-6 can substantiate this. However, with the long chronic exposure to TGF-β (right panel) may lead to a reduction in SMAD3 and with the IL-6 and Th17 cells may cause phenotypic changes in the remodelling process characterised by proliferation and migration in pulmonary smooth muscles and endothelial cells (see text).

Angeli et al noticed that schistosoma eggs selectively induce the synthesis of IL-6 in pulmonary microvascular endothelial cells and may reduce the inflammatory reaction^238^. Graham et al^239^ noticed that schistosoma-induced pulmonary hypertension in a model which lacked IL-6 signalling (at the level of either IL-6 or blocking STAT3) promoted the remodelling of the endothelial layer after *S. mansoni* exposure. This was attributed to possible effects of other inflammatory profile like enhancement of Th1 and Th2, which is in schistosoma-induced pulmonary hypertension and not manifested in chronic hypoxia model. At the same time, it decreased smooth muscle remodelling and increased in mice treated with a STAT3 inhibitor. The role of IL-6 may be related to the observations that it promotes the production of TGF-β1 probably by activation of macrophages and mast cells^240^. which consequently, promotes human pulmonary muscular layer differentiation into contractile smooth muscle-like cells^241^, and later , as per Zabini et al findings^242^ (Figure 10), the production and colocalization of IL-6, which is also is known to be secreted by macrophages and other cell types, such as mast cells^243^, contributing to further remodelling^212^.

These complex mechanisms are in concordance with many recent findings^233,242^. Furthermore, IL-21 was noticed to downstream the aggressive role of IL-6-signalling^244^ (Figure 10).

#### Transforming growth factor β

Transforming growth factor β (TGF-β) is a pleiotropic cytokine, which regulates a broad range of cellular processes, such as differentiation, proliferation, migration, apoptosis vascular homeostasis, and can influence both acute and chronic inflammation^245–247^. TGF-β signalling in pulmonary vascular remodelling is also mediated via several inflammatory mediators, including IL-1 and IL-6^233^ , with IL-6 appearing to play a key role, as documented in monocrotaline and chronic hypoxia rat models of models^248^.

IL-6 (induces cell signalling via various types of receptors specific for TGFβ which, through the activation of Janus kinases (JAK), lead to the activation of SMAD proteins (mainly SMAD2 and SMAD3) through phosphorylation^249^. TGF-β signal transduction has multiple transcriptional and non-transcriptional targets of target gene expression which can be influenced by other signalling pathways ^250^. Loss of BMPR-II (bone morphogenetic protein receptor 2) signalling contributes to the increase in signalling via TGF-β. A long line of research has shown that activation of the TGF-β system stimulates vasculogenesis, including intimal hyperplasia and medial smooth-muscle growth^251,252^.

TGF-β has been noted to contribute to pulmonary vascular pathology; but the complexity of the TGF-β signalling mechanism is far from being fully understood and some findings are counterintuitive. It was occasionally observed that the TGF-β signalling mechanism might be impaired, and a significant decrease in pulmonary SMAD3 expression was detected in pulmonary hypertension animal models as well as in patients^253–255^, suggesting a decrease in the activity of the canonical TGF-β pathway^242^. This conundrum of TGF-β has been recently investigated by Zabini and colleagues^242^ (Figure 10), where perpetual stimulation with TGF-β can down regulate the canonical TGF-β signalling, thus downstream of SMAD3 in the pulmonary vasculature. Reduced SMAD3 expression favours proliferation and migration in pulmonary smooth muscle and endothelial cells. Thus, initial endothelial cell injury may trigger pulmonary smooth muscle cell differentiation to a contractile proliferative phenotype. Later, progress of the inflammation and the reduction of SMAD3 leads to the development of a synthetic phenotype, which acquires migratory capacities. This causes an increase in vascular wall thickness, muscularization of small pulmonary arterioles, progressive excessive endothelial cell growth, and apoptosis-resistant phenotype^233,242,256–258^.

#### Vascular endothelial growth factor

Vascular endothelial growth factor (VEGF) is a key immune regulator is which is produced by Th2 inflammation and can itself contribute to Th2 pulmonary responses in a murine model of schistosomiasis-induced pulmonary hypertension^211^. VEGF receptor blockade partially suppressed the levels of the Th2 inflammatory cytokines interleukin (IL-4 and IL-13) in both the lung and the liver after *S. mansoni* exposure and suppressed pulmonary vascular remodelling^211^ illustrating the role of VEGF schistosomiasis-induced vascular inflammation and remodelling.

#### Interleukin 10

Interleukin 10 (IL-10) is a multifunctional cytokine, which is produced by CD4 T lymphocytes and macrophages. It can block NF *κ*B activity, and is involved in the regulation of the JAK-STAT signalling pathway. It has a major regulatory anti-inflammatory function. It down regulates Th1 cytokines by suppressing their excessive response and inhibits the synthesis of pro-inflammatory cytokines such as IFNγ, IL-2, IL-3, TNF *α*^140^. It also enhances B cell survival, proliferation, and antibody production, thus reducing an allergic reaction and immunopathology in many persistent infections, like in schistosomiasis^259,260^. Thus IL-10 plays a role in reducing morbidity and prolong survival in schistosomiasis^11,260,261^.

The plasma level of IL-10 is elevated with IL-6, along with TNF-α and IL-10 in non-fibrotic groups, but low IL-10 levels were associated with moderate/severe hepatic fibrosis - while IL-13 was raised in this group ^11^. These findings reveal the central regulatory role of IL-10 in the pathogenesis of schistosomiasis by supressing excessive type 1 and type 2 cytokine responses^261^. Ito et al, demonstrated that injections of IL-10 reduced the mean pulmonary arterial pressure and remodelling in monocrotaline pulmonary hypertension models in rats and inhibits the proliferation of cultured human pulmonary artery smooth muscle cells^262^.

In mice infected with *schistosoma ,* IL-10 levels increase in a time-dependent manner, in parallel with vascular remodelling^92^. Some investigators notice that IL-10 did not have direct antiproliferative effects on pulmonary artery smooth muscle, but HO-1 and CO may contribute to the anti-proliferative effect, which points to the necessity to decipher the role of the IL-10 pathway in the protection of pulmonary vascular diseases development^263^.

Elevated levels of IL-10 are found in patients with pulmonary arterial hypertension^264^. Patients under a pulmonary arterial hypertension target therapy with prostacyclin agonists showed higher levels of IL-10 compared to patients without such therapy^118^.

#### Interleukin 5

Interleukin 5 (IL-5) is an interleukin produced by type-2 T helper cells. IL-5 plays an essential role in recruiting the maturation of eosinophils to the site of antigen deposition^265,266^. It also play role in the regulation of other cytokines like IL-13^124,130^. With respect to pulmonary arterial hypertension, the effect of IL-5 may be similar to that of IL-4, and the severity of pulmonary arterial muscularization correlates with IL-5–expressing T cells^193^.

#### Interleukin -21

Interleukin (IL-21) is produced mostly by activated CD4^+^ T cells. It facilitates the differentiation and functional activity of the T helper cells, in particular the differentiation to Th17 cells (Box 3). IL-21 has pleiotropic effects on the proliferation, differentiation of B, T, natural killer, and dendritic cells. It also supporting the development of Th2 responses^267^, by increasing IL-4 and IL-13 receptor expression on macrophages and enhances the development of alternatively activated macrophages - which therefore, give it a potential role in inducing proliferation of primary pulmonary artery smooth muscle cells and profibrotic effects in the lung exposed to *S. mansoni*^267^; but this has not been studied systematically yet.

#### Interleukin 17

Interleukin 17 (IL-17) is a pro-inflammatory cytokine produced by a group of T helper 17 (Th17 cells) (Box 3). It has its own receptors in three forms referred to as IL17RA, IL17RB, and IL17RC. The signalling cascades results in induction of chemokines that activate various proinflammatory and inflammatory reactions and mediate many immune/autoimmune related diseases like rheumatoid arthritis, asthma, multiple sclerosis, psoriasis, and transplant rejection. IL-17 plays an essential role in inflammatory cells recruitment and accelerates inflammatory progression and thus can promote the granuloma formation and size in schistosomiasis^268,269^. This was opposed by IL-22^268^.

Experimental studies in animals have suggested Th17 promotes hypoxia-induced pulmonary hypertension^244,270^. Patients with idiopathic pulmonary arterial hypertension expressed a higher level of IL-17 and Th17 cells^271^. The role of IL-17 and Th17 in schistosomiasis pulmonary hypertension is still far from being systematically evaluated.

BOX 3 T helper 17 cells (Th17) are a subset of pro-inflammatory T helper cells defined by their production of interleukin 17 (IL-17). Naive CD4 T cells become activated when exposed to specific antigens differentiate into several possible T helper (Th1, Th2 , Th17 and Treg) cell subsets. This differentiation depends on a number of factors including antigen-presenting cells, cytokines and co-stimulatory molecules^276,277^. Th17 cells secrete the inflammatory cytokines IL-17 and IL-22, and their differentiation requires the presence of TGF-β, IL-6, IL-21 and IL-23^276–281^. The key factors in the differentiation of Th17 cells are signal transducer and the activator of transcription 3 (STAT3) and retinoic acid receptor-related orphan receptors gamma (RORγ) and alpha (ROR α)^282^. Further, Th17 cells inhibit Treg differentiation. Th17 cells are important in the control of extracellular and fungal pathogens and contribute to immunopathology in certain autoimmune diseases

#### Resistin-like molecule α

Resistin-like molecule α (RELM-α), also referred to as inflammatory zone 1 (FIZZ1), hypoxia-induced mitogenic factor (HIMF), or Retnla (resistin-like molecule alpha), is a cytokine produced during hypoxia in the lung^272,273^. RELM-α with HIMF, and the transcription factor HIF-2α, were temporally and spatially expressed in the developing lung and hypoxia. RELM-α was shown to exert an anti-inflammatory effect in parasite-induced Th2 responses and thus primarily functions as a regulatory molecule during helminth infection^274^. It regulates apoptosis products in allergen- and parasite-associated Th2 responses^275^.

## The Role of Co-Infection

Careful analysis of the published data suggests that the estimated prevalence of HIV-associated pulmonary arterial hypertension ranges from 0.4–5% globally^276^. It has been reported that many patients with HIV infection in Africa had co-infection with schistosomiasis^277–280^. A study in rural Zimbabwe reported that 57% of HIV patients were co-infected with *S. mansoni*, and that treatment of schistosomiasis could reduce the rate of HIV viral replication and increase CD4^+^ T cell count in the co-infected host^281^.

HIV viral proteins may enhance inflammatory reactions, mediate endothelial cell injury, and promote the remodelling process^276^. Co-infection with HIV is proposed to create an unusual complex inflammatory response, but biologically plausible circumstance, that would allow for severe and rapidly progressive pulmonary arterial hypertension. The inflammatory response due to schistosomes as described above can also alter the HIV infection. The release of cytokines and chemokines such as IL-6, or increased levels of vasoactive peptides like asymmetrical dimethylarginine, a well-known endogenous inhibitor of endothelial nitric oxide synthase, thus promotes endothelial dysfunction and vascular smooth muscle cell proliferation^282–284^. HIV viral proteins like Nef and Tat proteins modulate the release of IL-2 and monocyte chemotactic protein-1 (MCP-1, also known as CCL2), which stimulate pulmonary vascular remodelling particularly in Schistosomiasis^111,285,286^.

In addition, *S. mansoni* also appears to influence HIV replication, cell-to-cell transmission of HIV-1, and HIV disease progression as indicated by lower CD4^+^ T cell counts when co-infection is present. Deworming of HIV-positive individuals living in endemic areas may have an impact on HIV-1 viral loads and the CD4^+^ T cell count^287,288^. Schistosomiasis can also impair the response to antiretroviral therapy among HIV-infected patients^289^. The details and clinical significance of co-infection have not yet been studied, but is due for careful evaluation considering its importance, particularly in Africa where dual HIV-schistosomiasis infection is most prevalent^276,290^.

## The Prevalence of Pulmonary Hypertension Secondary to Schistosomiasis

The real prevalence of pulmonary vascular diseases caused by schistosoma infection is unknown. One of the earliest report of the presence of schistosoma eggs in the lungs of African natives was in 1885^291^. After that there were several reports from Egypt in the first half of the twentieth century, describing pathological and clinical manifestations of pulmonary vascular diseases secondary to infection by both *S. mansoni* and *S. haematobium* ^74,82,85,86,292^.

**Figure 11.**
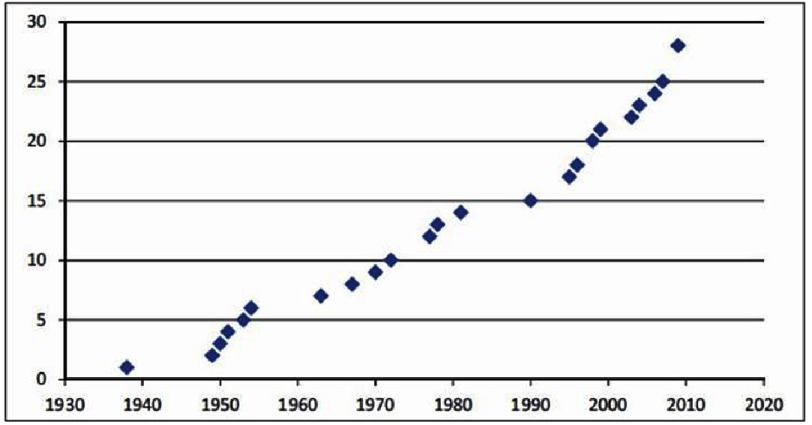
Number of studies (cumulative) assessing prevalence by year, showing a relative gap in research in pulmonary hypertension due to schistosomiasis from 1982 to 1995 and an increase since 1995 (from 293).

In the second half of the twentieth century, reports of many small series appeared in the literature, this time mainly from Brazil and occasionally a few cases from Africa and more recently, China (Figure 11). In these studies, the prevalence ranged from 7.7 to 33%^94,293–295^.

Barbosa et al, ^296^ examining 246 patients of a highly endemic area for schistosomiasis in Brazil, using echo and Doppler cardiograph, found pulmonary hypertension in 16% of the individuals with no case of cor pulmonale

Recently, more carefully controlled methodological studies were conducted in patients with hepatosplenic schistosomiasis and liver fibrosis in Brazil, and found that that 7.7–10.7% were diagnosed with pulmonary hypertension^46,94,294^. It is, however, difficult to estimate the real prevalence of pulmonary vascular diseases, worldwide, as it depends on many factors like the geographical distribution, public health status, difference in the antigenicity, and progress of the pathological changes. It is suggested from our experimental observation that the changes in the pulmonary vasculature after schistosoma infection are far more common, but may not always be associated with significant increases in the total vascular resistance, or clinical manifestation of pulmonary hypertension^90^.

## Acute Pulmonary Schistosomiasis (Katayama Syndrome)

The pulmonary involvement of schistosomiasis can be as early as the first exposure to infection^56^; causing a usually mild clinical condition called “Acute schistosomiasis”, or Katayama syndrome^297^. It is common in those infected with any schistosomiasis species for the first time; such as travellers or immigrants to schistosoma-endemic regions^3,298,299^. Historical accounts of Katayama disease date back to the discovery of S*. Japonicum* in Japan in 1904 (Figure 2). The disease was named after an area where it was endemic, Katayama district in Hiroshima prefecture, which was an endemic area in Japan until 1980^300^. The condition was also recorded historically, when – during the Napoleonic invasion of Egypt at the end of the 18th century – 35,000 French soldiers suffered from outbreaks of abdominal pain, fever, and occasionally haematuria. It is now believed to be due to acute schistosomiasis^301,302^.

The initial acute immunological response to schistosomiasis occurs following penetration of the skin by the cercariae, leaving an itchy rash known as cercarial dermatitis, or “swimmers itch”, within a few hours^6,299^. This condition can occur with all species of schistosoma, including those in which humans are not a natural host, such as the species that infect birds. Disease onset appears to be related to migrating schistosomula and egg deposition; thought to be an immune complex-mediated hypersensitivity reaction.

Katayama syndrome usually presents 4–8 weeks after water contact exposure with a combination of symptoms such as; general malaise, nocturnal low-grade fever, cough, myalgia, headache, wheezing, dyspnea, tender hepatomegaly, and gastrointestinal disturbances such as diarrhoea and abdominal pain^298,303^.

Chest x-rays show widespread transitory nonspecific infiltrates are often seen. Almost all cases have eosinophilia^304,305^. Other clinical manifestations include myocarditis^306^ and neurological involvement. Cerebral schistosomiasis may manifest as an acute or sub-acute encephalitic-like syndrome, as a slowly growing inflammatory pseudotumor with mass effect, or as a stroke syndrome^307^. Acute schistosomiasis was also reported during pregnancy with a trend of lower birth weights observed in the infants of the pregnant patients who were not treated^308^.

Acute schistosomiasis usually dissipates within several weeks, although respiratory complaints may continue for months^298^. Patients respond well to regimens of praziquantel with and without steroids. Artemisinin treatment given early after exposure may decrease the risk of the syndrome. 26% of patients who were initially asymptomatic developed chronic schistosomiasis within 3 years of exposure^298,307^.

## The Clinical Presentations of Pulmonary Hypertension Secondary to Schistosomiasis

Pulmonary vascular disease secondary to schistosomiasis is more common in endemic areas and may not always produce clinical symptoms^90^. It is only on repeated infection that severe remodelling of the vessels through a high antigenicity load of eggs from the schistosoma can produce pulmonary hypertension, and consequently right heart failure^90^.

**Figure 12.**
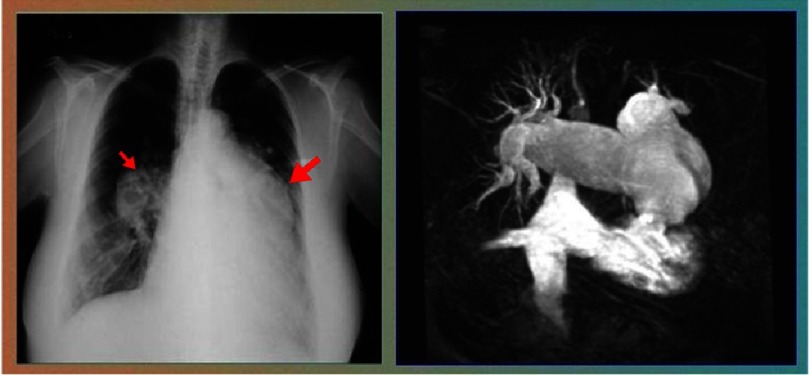
A chest radiograph of a 52-year old man with pulmonary hypertension due to schistosomiasis. The X ray to the left and right show the dilatation of left and right pulmonary arteries (arrows). the right panel showing the characteristic dilation of both main right and left pulmonary artery in patients with schistosomiasis pulmonary hypertension (images: courtesy of Dr Angela Bandeira).

**Figure 13.**
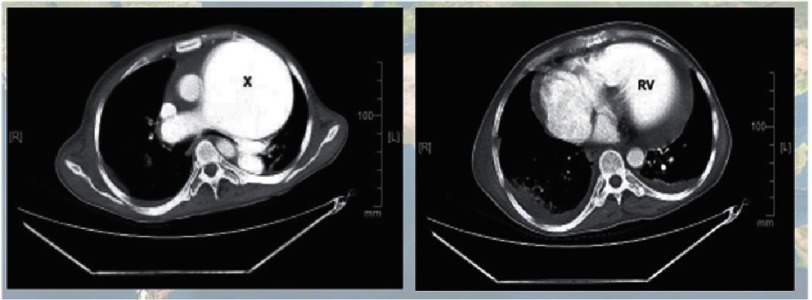
CT scan of patient with pulmonary hypertension due to schistosomiasis. The left panel shows severe pulmonary artery dilatation (X), and in the right panel shows the right ventricular dilatation and hypertrophy. (Images: courtesy of Dr T. Safwat).

The clinical presentation shows signs and symptoms that are not distinguishable from another form of pulmonary arterial hypertension; such as dyspnoea on exertion, anaemia, fatigue, weakness, cough, giddiness fainting, and exercise intolerance. Physical examination may reveal a prominent pulmonic component second heart sound, right ventricular heave, and digital clubbing. Radiographs may reveal cardiomegaly, particularly dilatation of the right ventricle and right atrium, and enlarged pulmonary trunk and arteries, with pruning of the distal vasculature. Clinical and radiological findings are similar to those associated with other causes of pulmonary hypertension. It was, however, demonstrated that pulmonary artery enlargement is more pronounced in schistosomiasis pulmonary hypertension than another form of pulmonary arterial hypertension; which may be a feature of schistosomiases pulmonary hypertension^309–311^ (Figure 12 and Figure 13). Few cases of giant pulmonary artery aneurysm in a patient with pulmonary arterial hypertension associated with schistosomiasis have been reported^312^ and may be complicated by pulmonary artery dissection^313^.

Electrocardiography typically shows right ventricular hypertrophy or strain, and right atrial enlargement. It may also reveal a right bundle branch block. Echocardiography demonstrates right ventricular dilatation, potentially compressing the left ventricle with septal bowing, usually accompanied by right atrial dilation, tricuspid valve regurgitation, and an increased pressure gradient across the tricuspid^296,314^.

It is important to rule out other causes of pulmonary hypertension to assess causality. Patients in endemic areas should suspect schistosomiasis as the cause of pulmonary hypertension, in particular with the presence of prehepatic portal hypertension^30^. It is, however, essential to perform right heart catheterization, to provide a direct measurement of the mean pulmonary arterial pressure, and assess the right ventricular function and pulmonary artery wedge pressure. It is worth mentioning that about 13% of patients with schistosomiasis pulmonary hypertension will also have compromised left ventricular function with the increase of the PAWP^315^. Only a small number of patients had significant vasodilatory response acute vasodilator challenge^316^.

There is no pathognomonic feature to diagnose schistosomiasis pulmonary hypertension. We do not yet have biomarkers or serological tests to help in the diagnosis^317^. Most centres depend on an empirical approach that reveals the presence of pulmonary hypertension in the absence of significant lung parenchymal disease (e.g., left ventricular dysfunction or chronic thromboembolic disease, associated with the presence of periportal fibrosis and/or left liver lobe enlargement associated with at least one of the following features: positive epidemiology, previous treatment of schistosomiasis or identification of eggs in stool or rectal biopsy^316,318,319^).

## Treatment of Schistosomiasis Associated with Pulmonary Arterial Hypertension

There are no clinical trials to confirm the application of any of the currently approved therapies for pulmonary arterial hypertension in schistosomiasis pulmonary hypertension. So far we only have observational data from various centres around the world. It has been conventional in many centres to treat these patients with the current treatment such as phosphodiesterase-5 inhibitors or endothelial receptor antagonists^320–323^. These observational small studies showed improvements of functional class, cardiac output and 6-min walking test distance (6MWT)^322^.

In a retrospective and prospective observational single centre study involving 102 patients with schistosomiasis pulmonary hypertension, it was suggested that patients receiving the current conventional pulmonary arterial hypertension therapy had better survival rates than a historical untreated group at 60 months (89.1% vs. 69.2% respectively)^324^.

It is important to determine if active schistosomiasis infection is present, as treatment with an anthelmintic drug, such as praziquantel, may be warranted. Praziquantel works on the schistosome calcium ion channels, causing paralysis in the contracted element of the parasite that facilitates detachment of adult worms from the host vascular wall, and ultimately leads to their death^325^. It has a cure rate of up to 90%. This may help to reduce the antigenic load from the parasite eggs, thus reducing the granuloma, and probably prevent further deterioration^83,326,327^.

Experimental studies showed that anti-schistosomal therapy reduces pulmonary vascular remodelling and, consequently, pulmonary hypertension^326^. However while this strategy may be useful in acute conditions, it may not be beneficial in chronic pulmonary hypertension, where studies suggest that pulmonary remodelling and clinical pulmonary hypertension may persist even after complete deworming and disappearance of eggs^104^.

There is however a small risk of using anti-schistosomal therapy, whereby the dead worm may cause pulmonary embolism with an abrupt increase in pulmonary pressure and development of acute cor pulmonale^104^. Managing other consequences of infection, such as the used of corticosteroids, may be useful, but it is uncertain whether they have any effect on the pulmonary vascular pathology.

Another important issue in the management of schistosomiasis is the frequent association of liver diseases, which necessitates closer monitoring of liver function, in particular when endothelial antagonists are used. Anticoagulation drugs in patients with schistosomiasis pulmonary hypertension should be considered with care, because the presence of portal hypertension and related complications such as oesophageal varices and the risk of bleeding^105,328,329^.

Furthermore, the surgical malmanagement of the oesophageal varices, like trans jugular intrahepatic Porto systemic shunt and distal splenorenal shunt, can increase the load on the right ventricle, increasing the risk of more shunting of eggs from the portal system^330^. Thus, increasing the antigenic load to the lung increases the risk of more granuloma and pulmonary vascular remodelling – leading to the risk of fatal pulmonary hypertension^105^. However, prognosis of this condition has not been systematically studied. Initial small observations showed a more benign clinical course than idiopathic pulmonary arterial hypertension^316^.
